# Uterine Mass and Menorrhagia: A Rare Presentation of Acute Myeloid Leukemia with Arduous Clinical Course

**DOI:** 10.4274/balkanmedj.2017.0941

**Published:** 2018-05-29

**Authors:** Kundan Mishra, Chandrasekaran Muralidaran, Aditya Jandial, B.R. Mittal, Subhash Varma

**Affiliations:** 1 Department of Internal Medicine, Postgraduate Institute of Medical Training and Research, Chandigarh, India; 2 Department of Pathology, Postgraduate Institute of Medical Training and Research, Chandigarh, India; 3 Department of Nuclear Medicine, Postgraduate Institute of Medical Training and Research, Chandigarh, India

A 36-year-old multiparous lady presented with complaints of menorrhagia and fatigue for three months. There was no history of bleeding from any other site, fever, lower abdominal pain, or abdominal distension. She was diagnosed with primary hypothyroidism five years back, and since then she had regularly been on a stable thyroxine dose (125 μg per day); there was no history of any other drug intake in the past. Surgical history was noteworthy for cesarean section ten years back. She was hemodynamically stable; no pallor, goiter, or petechial spots observed on general physical examination. Systemic examination was inconspicuous. Hemogram (hemoglobin 128 g/L; total leucocyte count 7.6x109/L; and platelet count 360x109/L), peripheral blood smear and coagulogram were normal. Urine for pregnancy test was negative; serum prolactin and thyrotrophin-stimulating hormone levels were normal. To rule out local genital lesion, a detailed pelvic examination (including speculum examination) was performed. The posterior lip of the cervix appeared bulky with a 2x1 cm reddish-pink, sessile, firm, nodular lesion arising from it. At this point, the possibility of cervical carcinoma versus benign cervical lesion (mucus retention cyst, ectropion, benign polyp, endometriosis, cervicitis, etc.) was considered. Brushed smear from the cervical lesion revealed high-grade dysplasia. Pelvic ultrasound revealed an ill-defined heterogeneous, hypoechoic soft tissue lesion involving the cervix; there was no vaginal or parametrial involvement. A total abdominal hysterectomy was performed. Surprisingly, histologic examination of the uterus revealed infiltration of muscle bundles, consisting of sheets of atypical cells reminiscent of lymphoid cells (Figure 1a). Immunohistochemistry showed strong myeloperoxidase positivity in the atypical cells, confirming a diagnosis of granulocytic sarcoma (Figure 1b). Bone marrow examination showed 22% blasts, which were strongly myeloperoxidase positive (Figure 2a) and were also CD34+ (Figure 2b) confirming the diagnosis of acute myeloid leukemia. Conventional cytogenetics revealed t(5:12) and trisomy 21 (Figure 2c); the acute myeloid leukemia molecular panel (FLT3, CEBPA, NPM1, C-kit, and BCR-ABL) was negative. A diagnosis of acute myeloid leukemia with recurrent cytogenetic abnormalities and extra-medullary granulocytic sarcoma of the uterus was made, and she was started on standard (7+3 regimen) induction therapy. However, the chemotherapy dose had to be abbreviated (5+2 regimen) in view of poor performance status and development of septic shock. Day 28 bone marrow examination suggested morphological remission. Due to the absence of a full match donor, she was planned for consolidation chemotherapy with high dose Cytarabine (three g/m^2^ twice daily on D1, D3, and D5). On day 31 of induction chemotherapy, she was noticed to have enlarged right cervical lymph nodes (level 3 and 4). Fine needle aspiration cytology revealed leukemic infiltration. Whole-body positron emission tomography computed tomography showed intense fludeoxyglucose uptake in right cervical, bilateral axillary, and pelvic lymph nodes (Figure 2d). She was started on salvage chemotherapy with high dose Cytarabine plus Mitoxantrone (high-dose AraC and Mitoxantrone protocol). However, she died on day 15 of chemotherapy due to septic shock. An informed consent for publication has been obtained from the patient.

Granulocytic sarcoma is an uncommon presenting manifestation of acute myeloid leukemia. In our experience, we have observed granulocytic sarcomas in unusual locations like orbit (1), mediastinum (2), intracardiac (3), and cervico-dorsal spine (4) causing Horner’s syndrome acute myeloid leukemia presenting as uterine granulocytic sarcoma is extremely rare. Presence of granulocytic sarcoma is known to be a poor prognostic marker in acute myeloid leukemia. Such patients generally respond poorly to the standard acute myeloid leukemia induction, as in our case, and they often require salvage chemotherapy and stem cell transplantation. Detection of granulocytic sarcoma may precede or concur with an acute myeloid leukemia diagnosis. Cervical carcinoma shall always remain the first possibility in any patient with such presentation, but there are a few important distinctive points that the index case highlights. The presence of a sessile cervical lesion on speculum examination, high-grade dysplasia on cytology, and a heterogeneous soft tissue cervical lesion on ultrasound prejudiced the diagnosis towards cervical carcinoma. Carrying out a punch biopsy from the cervical lesion may have established the diagnosis of myeloid sarcoma before hysterectomy and averted poor performance status due to surgery. Although cervical cytology is an effective and convenient screening tool, a biopsy must be regarded as the standard diagnostic procedure; at least in all visible cervical lesions. Secondly, this case reiterates the fact that presence of granulocytic sarcoma in acute myeloid leukemia almost always confers a poor prognosis, irrespective of the location. Thirdly, the role of fludeoxyglucose-positron emission tomography scan in granulocytic sarcoma shall be useful in unmasking the asymptomatic disease in the rest of the body at baseline, modification of treatment strategies depending upon the involved site, and assessment of response to treatment (5). Lastly, it may not be necessary that the pace of granulocytic sarcoma will always match the bone marrow disease evolution.

## Figures and Tables

**Figure 1:**
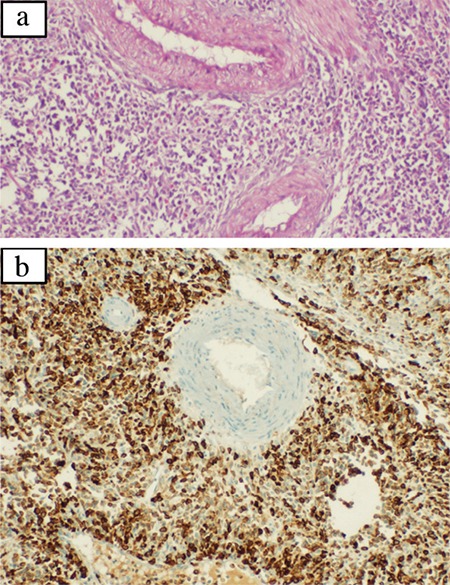
Microphotograph showing sheets of atypical lymphoid cells seen infiltrating in between the muscle bundles and also seen around the muscular arteries (H&E, x200) (a), microphotograph highlighting strong myeloperoxidase positivity in the atypical lymphoid cells confirming it be a granulocytic sarcoma (myeloperoxidase immunohistochemistry, x200) (b).

**Figure 2:**
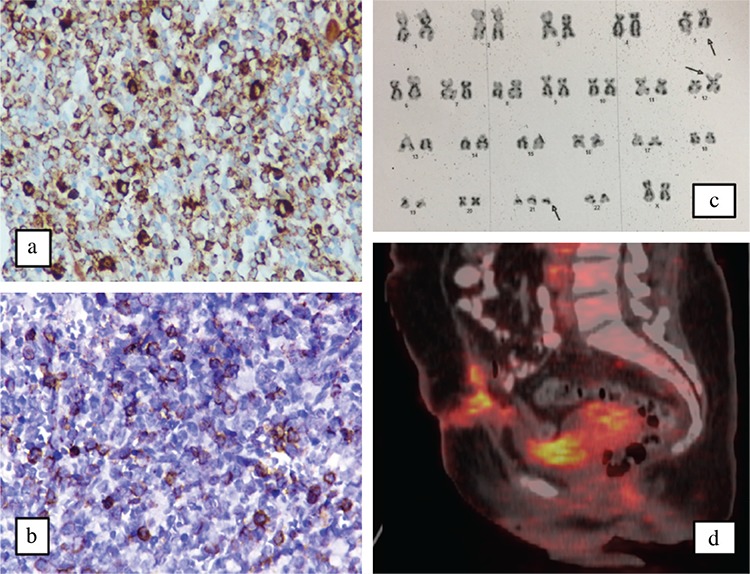
Microphotograph highlighting strong myeloperoxidase positivity in the blast cells confirming it be a acute myeloid leukemia (myeloperoxidase immunohistochemistry) (a), microphotograph highlighting strong CD34 positivity in the blast cells (b), Photograph of cytogenetic studies shoeing t(5:12) and trisomy 21 (c), positron emission tomography-computed tomography showing intense fludeoxyglucose avid lesions involving retroperitonium and pelvis (d).
